# Quality indicators for safe and effective medication use in long‐term care facilities: A modified Delphi study

**DOI:** 10.1002/bcp.70531

**Published:** 2026-04-02

**Authors:** Daria S. Gutteridge, Sara Javanparast, Annabel H. Calder, Gillian E. Caughey, Andrew C. Stafford, Gregory M. Peterson, Maria C. Inacio, Peter D. Hibbert, Elizabeth Manias, Jodie B. Hillen, Janet K. Sluggett

**Affiliations:** ^1^ UniSA Allied Health and Human Performance University of South Australia Adelaide South Australia Australia; ^2^ Registry of Senior Australians Research Centre South Australian Health and Medical Research Institute Adelaide South Australia Australia; ^3^ Registry of Senior Australians Research Centre, Caring Futures Institute, College of Nursing and Health Sciences Flinders University Bedford Park South Australia Australia; ^4^ Curtin Medical School and enAble Institute, Faculty of Health Sciences Curtin University Perth Western Australia Australia; ^5^ School of Pharmacy and Pharmacology University of Tasmania Hobart Tasmania Australia; ^6^ Australian Institute of Health Innovation Macquarie University Sydney New South Wales Australia; ^7^ Monash Nursing and Midwifery Monash University Clayton Victoria Australia; ^8^ School of Nursing and Health Science Flinders University Bedford Park South Australia Australia; ^9^ South Australian Health and Medical Research Institute Adelaide South Australia Australia

**Keywords:** monitoring, nursing home, prescribing, quality use of medicines

## Abstract

**Aim:**

This modified Delphi study aimed to achieve expert agreement on quality indicators (QIs) suitable for application at the population level, to evaluate quality use of medications and pharmacist services in long‐term care facilities (LTCFs).

**Methods:**

We conducted a two‐round modified online Delphi study with a multidisciplinary panel of Australian subject matter experts (*n* = 25). Experts rated 58 QIs, identified in a recent systematic review, on three criteria (importance, feasibility and amenability to change by an on‐site pharmacist) using a 9‐point Likert scale. A QI was selected if it reached agreement between expert members (defined as a disagreement index of ≤1) with a high median score (≥7 on the Likert scale) across all three criteria.

**Results:**

Twenty‐five experts completed the first Delphi round, and 24 completed the second round. Overall, high scores with agreement were obtained for 45 QIs (78%) for importance, 27 QIs (47%) for feasibility and 25 QIs (43%) for amenability to change. Seventeen of the 58 QIs received high scores in agreement across all three criteria and were selected, covering: multidisciplinary clinical care (*n* = 7 QIs), clinical governance (*n* = 5), medication‐specific issues (*n* = 3) and end‐of‐life care (*n* = 2).

**Conclusion:**

The identified QIs provide a valuable foundation to capture and monitor the complexity of medication management, including on‐site pharmacist services, in LTCFs. Subject to future testing and research, the expert‐prioritized QIs could help optimize medication‐related quality of care efforts and improve outcomes for residents in LTCFs.

**What is already known about this subject**

- A quality monitoring programme that assesses a broad range of medication‐related activities and pharmacist services in long‐term care facilities (LTCFs) is required to drive care quality and outcomes, and inform the provision of pharmacist services.
- Existing medication‐ and pharmacist‐related quality indicators need to be assessed for suitability.

**What this study adds*:***

- This study identified 17 quality indicators that are important, feasible to measure on a population‐level and amenable to change by an on‐site LTCF pharmacist, to monitor and evaluate safe and effective medication use in LTCFs.
- Indicators identified cover medication review and reconciliation activities, clinical governance and medication‐specific indicators including anticipatory medications.

## 1 INTRODUCTION

Long‐term care facilities (LTCFs), also known as nursing homes, care homes or residential aged care facilities, provide personal and nursing care and palliative care services to older people who can no longer reside at home.^1^ Residents of LTCFs often have complex health needs, with multimorbidity and frailty, and may require treatment with multiple medications.^2^ The use of high‐risk medications, such as opioids and antipsychotics, is highly prevalent and up to 91% of residents are exposed to polypharmacy and over 95% experience at least one medication‐related problem during their LTCF stay.^3^, ^4^, ^5^ These factors place residents at risk of medication errors and adverse health outcomes, such as falls and hospitalizations.^6^, ^7^ It is estimated that up to two thirds of medication‐related hospitalizations are potentially preventable.^8^, ^9^ Hence, having appropriate medication management systems in place is crucial to reduce medication‐induced harm and support safe and effective medication use in LTCFs.

Multiple initiatives have been implemented internationally, often led by pharmacists, to improve medication management in LTCFs.^5^ These initiatives include individual‐level clinical activities, such as comprehensive medication reviews, and provision of facility‐level activities such as staff education and interprofessional clinical governance activities.^5^, ^10^, ^11^ Multidisciplinary medication advisory committees (MACs) are an Australian‐specific initiative in LTCFs^5^, ^12^ that advise on the development, implementation and monitoring of medication management policies, procedures and outcomes.^13^ However, in many LTCFs, resident interactions and interprofessional communication involving clinical pharmacists remains limited, thereby constraining a holistic, patient‐centred approach to medication management.^5^ In Australia, the Government‐funded Aged Care Onsite Pharmacist (ACOP) programme was introduced in LTCFs^14^ in 2024 to facilitate interdisciplinary collaboration and communication, provide individualized clinical services and contribute to clinical governance aimed at supporting safe and effective care to optimize health outcomes among residents.^5^, ^15^ This new model of care delivery, which will cost the Australian Government $350 million AUD over 4 years, is promising, but currently no formal evaluation of this programme is in place.^16^, ^17^

Given the relative novelty of integrating clinical pharmacist services on‐site in LTCFs, there has been limited focus internationally on monitoring the impact of clinical pharmacist services in LTCFs.^18^ To appropriately evaluation emerging models, relevant population‐level quality indicators (QIs) are needed to monitor, evaluate and inform improvements in care.^19^ QIs quantitatively measure performance, including the structure, processes and outcomes of care.^20^ Although many QIs have been developed to assess medication use in LTCFs,^18^, ^21^ not all are suitable for routine population‐level monitoring. To be applicable at the population level (i.e., national and state or regional level), QIs must be feasible, which depends on the availability of reliable and easily collectible data, and should be amenable to change by healthcare practices.^19^, ^22^, ^23^ However, existing population‐level medication‐related QIs often focus on medication‐specific concepts, overlooking broader aspects of pharmacist services and multidisciplinary care in LTCFs.^18^, ^19^ For instance, the National Mandatory Quality Indicator programme in Australian LTCFs^24^ currently includes two medication management QIs, prevalence of (i) antipsychotic use and (ii) polypharmacy. This is consistent with the monitoring programmes in LTCFs in other high and middle‐income countries, with polypharmacy being the most commonly assessed medication‐related QI, currently implemented in 6 of 29 evaluated LTCF monitoring systems.^19^, ^25^

Given the growth in pharmacist‐led initiatives internationally to improve medication management in LTCFs, a tailored quality monitoring programme to monitor the impact of these unique and promising services on resident's outcomes is required.^17^ This study, as part of the PHarmacists Actioning Rational use of Medicines in Aged Care (PHARMA‐Care) project, aimed to identify suitable QIs to assess the safe and effective use of medications and pharmacists' services in LTCFs, with indicators intended for facility level measurement and national reporting. Building on a recent systematic review,^18^ which identified a broad range of potential QIs, this Delphi study engaged with a diverse panel of aged care experts to gather expert input and identify agreement on QIs that are appropriate for population‐level monitoring, with indicators intended for facility‐level measurement and national reporting.

## 2 METHODS

### 2.1 Development of an initial set of quality indicators

**FIGURE 1:**
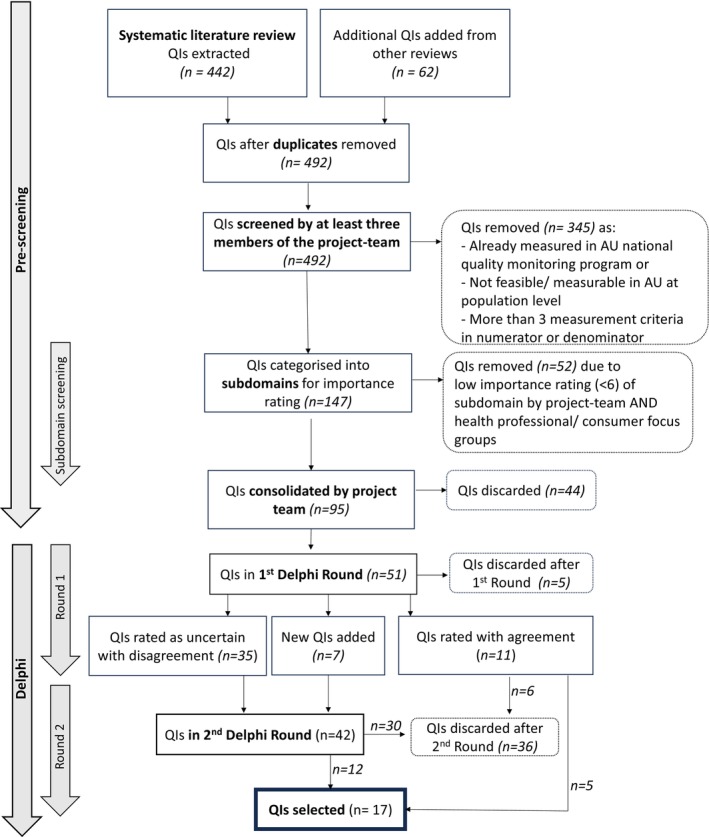
Quality indicator (QI) selection flow diagram.

The QIs selected for the first Delphi round were based on a recent systematic review,^18^ that identified 442 QIs to monitor the safe and effective use of medications in LTCFs and in‐home aged care services (Figure 1). Additional relevant studies, outside the systematic review's search date range were also screened, identifying 62 additional QIs. From these 504 QIs, the following were excluded (*n* = 345): (i) duplicates; (ii) QIs related to polypharmacy or antipsychotic use, as these are already monitored nationally in Australia^24^; (iii) QIs considered neither feasible nor measurable at a population level within Australia; and (iii) QIs that contained more than three measurement criteria in the numerator or denominator as these generally lack feasibility in LTCFs.^26^ The remaining 147 QIs were then grouped into themes and subthemes, based on the initial systematic review.^18^ Nine people from the project team, with expertise in pharmacy, epidemiology, public health, aged care and health service monitoring, were invited to rate the subthemes on importance using a 9‐point Likert scale. QIs that fell into subthemes with a median importance rating of 5 or below were subsequently removed, and QIs within a subtheme with a median rating of 7–9 (high importance) progressed to the next stage. Subthemes with a median score of 6 were further discussed in two half‐day workshops with health professionals and consumers to identify domains and/or QIs that should be included. The project team consolidated the remaining QIs that assessed similar concepts and refined the QI definitions, numerators and denominators to the Australian LTCF context, leading to a list of 51 individual QIs that progressed to the first Delphi Round. The 51 QIs were then categorized into six domains informed by a policy review^13^ and qualitative study,^16^ that were presented in the Delphi in the following order: (i) governance and leadership, (ii) multidisciplinary clinical care and pharmacist services, (iii) medication‐specific indicators, (iv) person‐centred care and communication, (v) staff education about safe and effective medication use and (vi) palliative and end of life medications.

### 2.2 Delphi study design

We used an online two‐round modified Delphi process, via the RAND/UCLA Appropriateness Method (RAM),^27^ to identify suitable QIs of high priority and followed the DELPHISTAR reporting guidelines.^28^ The RAM was developed to identify agreement among experts and is a validated method for developing and selecting QIs.^27^ Both Delphi rounds were hosted on the online survey software *Surveylet* (Calibrum International, USA, calibrum.com), which panellists used to independently rate the indicators. For each round, participants were presented with an introduction page and video, before they progressed to the QI rating. Each QI was presented with a description, a numerator and a denominator, QI type based on the Donabedian Framework^29^ (i.e., structure, process and outcome) and additional background information. At the end of the survey, participants completed a brief demographic questionnaire and were provided with the option to receive a gift voucher. All panellists received an individualized survey link and provided informed consent for each round, via an online declaration in Calibrum. Ethics approval was provided by the University of South Australia Human Research Ethics Committee (ID:206207).

### 2.3 Selection of expert panel

Individuals with a range of different professional backgrounds and extensive knowledge and experience in medication management, practices, care quality and measurement within Australian LTCFs were considered as experts and invited to participate. Panellists were required to be ≥18 years, proficient in English, have at least 2 years of experience in medication management in Australian LTCFs, and be available to complete both Delphi rounds. A total of 57 experts were identified by the project team and invited to participate.

### 2.4 Rating criteria, definition of agreement and decision rules

#### 2.4.1 Round 1

The first round was open for 3 weeks in October 2024. Panellists rated each QI on the three criteria (importance, feasibility of data collection/reporting, and amenability to change by an on‐site pharmacist) outlined in Table 1, on a 9‐point Likert scale, with higher scores reflecting higher performance of the indicator. Panellists were given the opportunity to provide comments on each QI and domain and for some QIs additional questions were presented (e.g., to rate the preferred measurement period). A content analysis of participants' written feedback was conducted to incorporate wording changes, additional background information and the addition of new QIs for the second round. Median scores for each rating criteria across individual QIs, as well as the level of agreement among the panellists, were calculated in accordance with the RAND/UCLA Appropriateness Method.^27^ Level of agreement was assessed via the disagreement index (DI), computed by dividing the interpercentile range (i.e., the difference between the 30th and 70th percentile) by the symmetry of the rating (i.e., interpercentile range adjusted for symmetry) with a DI ≤ 1 indicating agreement. Decision rules (outlined in Table 2) were based on the median QI score and the agreement level across each of the three rating criteria. After the first round, QIs with a median score <7 for all three rating criteria were discarded. QIs with suggested wording changes and QIs that showed disagreement (DI > 1) in one of the three rating criteria but had a median rating of 7 or above in at least one of the two other rating criteria were re‐evaluated in Round 2.

**TABLE 1:** Rating criteria for quality indicators.

Criteria	Description	Rating levels
Importance of QI	Is the concept important to measure? Is the measure evidence‐based? Is there opportunity for improvement?	Low = 1–3 Moderate = 4–6 High = 7–9
Feasibility of data collection and reporting in LTCFs	Is the data collection and implementation feasible? Is there data that is readily available? Can the data be collected with minimal burden?	Low = 1–3 Moderate = 4–6 High = 7–9
Amenability of the QI to change by an onsite pharmacist	Can the concept being measured by the QI be influenced by Onsite Pharmacists activities?	Low = 1–3 Moderate = 4–6 High = 7–9

*Note*: Quality indicator, importance and feasibility criteria are based on the US National Quality Forum. The rating criteria, *importance of QI*, was set up as a forced choice response.

Abbreviation: QI, quality indicator.

**TABLE 2:** Classification and decision rules for rated quality indicators (QIs) in the two Delphi rounds.

Round 1	
Agreement	If DI ≤ 1 across all three rating criteria. QI was put aside
Disagreement	If there was disagreement (DI > 1) in one or more of the three rating criteria. The QI was moved to the 2nd Round
Adjusted for Round 2	If panellists suggested changes to the wording of the QI
Discard	If median score < 7 for importance AND amenability AND feasibility
Add	Additional QIs that were suggested by panellists
Round 2	
Selected	Median score ≥7 with agreement (DI ≤ 1, or if DI > 1 at least 70% of panellists have given it a high score (≥7)). This needs to apply in all three rating criteria for the QI to be selected
Not selected	Median score <7 and/or disagreement (DI > 1 with less than 70% of panellist giving it a high score (≥7)) in at least one of the three rating criteria

Abbreviation: DI, disagreement index.

#### 2.4.2 Round 2

The second Delphi round was open for 5 weeks (December 2024 to January 2025) and followed the same structure as the first round. Panellists re‐rated QIs that did not reach agreement in the first round, as well as newly added QIs (see Figure 1). For each QI, panel members were provided with their own individual ratings from the first round, together with a summary of the group's ratings, consisting of the median scores and deidentified comments from panellists. The agreement level from Round 1 and appropriateness (i.e., agreement and a median score ≥7) for each rating criteria were displayed. Any changes made to the QI (e.g., wording and background information) and newly added QIs were displayed in a different font colour. After the round closed, the median score for each rating criteria across individual indicators and the level of agreement were re‐calculated, and a content analysis was conducted on panellists' comments. The percentage of panellists who gave the QI a high rating score (i.e., score ≥7) for each of the three rating criteria was also determined.

#### 2.4.3 Selection of the final QI set

QIs were selected based on their final median score and agreement level across the three rating criteria. Agreement was primarily based on the DI. However, if the DI exceeded one, but ≥70% of panellists assigned a score of ≥7, the QI was still considered to have reached agreement, as in this situation the DI indicates disagreement within a high score range (7–9). A QI was selected and considered useful to measure medication safety and effectiveness and/or pharmacist services in Australian LTCFs, if it achieved a median score of at least 7 on each rating criterion with agreement. Table 4 indicates via colour, whether the selection criteria were met, with green indicating a high score (≥7) with agreement (DI ≤ 1 = dark green; or DI > 1 with ≥70% of rating = bright green) and red indicating a low score and/or disagreement.

## 3 RESULTS

### 3.1 Expert panel

Of the 57 experts invited, 27 agreed to participate; of these, 25 completed Round 1 and 24 completed Round 2. The panellist characteristics are displayed in Table 3.

**TABLE 3:** Expert panel member's (*n* = 25) characteristics.

	*N* (%)
Gender	
Female	16 (64)
Male	9 (36)
Australia state/territory of residence	
South Australia	12 (48)
New South Wales	5 (20)
Tasmania	4 (16)
Victoria	2 (8)
Other	2 (8)
Profession^a^	
Registered pharmacist	12 (48)
Academic/researcher	10 (40)
Medical practitioner	5 (20)
Registered nurse	3 (12)
Policymaker	3 (12)
Other	2 (8)
Level of expertise in primary profession	
>5 to 10 years	2 (8)
>10 to 20 years	6 (24)
>20 years	17 (68)
Number of years of experience in aged care	
>5 to 10 years	7 (28)
>10 to 20 years	7 (28)
>20 years	11 (44)
Medication advisory committee member	
Current member	6 (25)
Past member	6 (25)
No	12 (50)

^a^ Some experts had multiple professions.

### 3.2 Round 1

There were 51 QIs included in Round 1 (process QIs *n* = 42, structure QIs *n* = 3 and outcome QIs *n* = 6) (Tables 4, 5 and S1). Based on the classification rules, five QIs were discarded due to low ratings for importance, amenability to change and feasibility. There were 11 QIs that reached agreement across the three rating criteria (Table 1, Figure 1), and of these, 5 received high ratings and were selected for the final QI set. Another 35 QIs were classified as uncertain due to disagreement in some or all rating criteria and were re‐evaluated in Round 2. Additional QI topics were suggested by panellists and after consulting existing literature, seven suitable QIs were added to Round 2. Panel members also had to select the most appropriate timeframe in which residents should receive at least one comprehensive medication review for QI 9 (multiple answers could be selected). Every 12 months was selected by 30%, every 6 months by 39%, every 3 months by 22% and other by 26% of panellists.

**TABLE 4:** Definition of quality indicators (QI) and illustration whether the selection criteria were met (green) for each of the three rating criteria.

QI	Definition	Importance	Feasibility	Amenability	Selected
QI 1	Percentage of LTCFS that participate in a MAC.^a^				Yes
QI 2	Percentage of LTCFs with a MAC that meets at least quarterly.				Yes
QI 3	Percentage of MACs which are multidisciplinary and include a pharmacist.				Yes
QI 4	Percentage of MACs that reviewed trends in use of high‐risk medicines.				No
QI 5	Percentage of MACs that have used the ‘MAC Audit tool and checklist’ to assess performance in the last 12 months.				Yes
QI 6	Percentage of MACs that reviewed education needs and plans in the last 12 months				Yes
QI 7	Percentage of care recipients for whom the pharmacist documents a best possible medicines history within 7 days of admission.				Yes
QI 8	Percentage of care recipients who receive a comprehensive medicines review within 7 days of admission.				Yes
QI 9	Percentage of care recipients who received a comprehensive medicines review in the last 6 months.^a^				Yes
QI 10	Percentage of care recipients who received a comprehensive medicines review in response to worsening health.				No
QI 11	Percentage of care recipients with a fall who receive a comprehensive medicines review within 7 days of that fall.				No
QI 12	Percentage of care recipients charted a psychotropic medicines (i.e., antidepressant, antipsychotic, hypnotic/anxiolytic) for regular administration who received a comprehensive medicines review within the last 3 months.^a^				Yes
QI 13	Percentage of care recipients with a high drug burden index who received a comprehensive medicines review.				No
QI 14	Percentage of care recipients experiencing a medication incident who were reviewed by the pharmacist within 7 days of the incident.^a^				Yes
QI 15	Percentage of comprehensive medicines reviews that included at least one personal exchange between the pharmacist and a nurse, as well as between the pharmacist and the care recipient or their family member/proxy.				No
QI 16	Percentage of care recipients who received a comprehensive medicines review where the pharmacist discussed the outcomes of the medicines review with the care recipient or family member/proxy.				No
QI 17	Percentage of recommendations made by the pharmacist during comprehensive medicines reviews that have been implemented.^a^				No
QI 18	Percentage of care recipients transferred/discharged out of the LTCF with a current medicines list provided at that time.				No
QI 19	Percentage of care recipients who received a comprehensive medicines review in response to changes in medicines use.				No
QI 20	Percentage of care recipients charted a new psychotropic medicine (i.e., antidepressant, antipsychotic and hypnotic/anxiolytic) who received a comprehensive medicines review within 6 weeks.				Yes
QI 21	Percentage of care recipients who received a comprehensive medicines review where the pharmacist discussed the recommendations from the medicines review with the care recipient's prescriber.				No
QI 22	Percentage of GP rounds attended by a pharmacist				No
QI 23	Percentage of family meetings/case conferences attended by a pharmacist.				No
QI 24	Percentage of pharmacist visits to the LTCF that were provided by the same pharmacist.				No
QI 25	Amount of time that the pharmacist is onsite in the LTCF.				Yes
QI 26	Percentage of care recipients who received an antimicrobial.^b^				No
QI 27	Number of antimicrobial treatment days per 1000 resident days.^b^				No
QI 28	Percentage of antimicrobial prescriptions with a documented indication for use.				No
QI 29	Percentage of antimicrobial prescriptions with a documented review or stop date.				No
QI 30	Percentage of care recipients, without a contraindication, who received the influenza vaccination over a 12‐month period.				Yes
QI 31	Percentage of care recipients who received an antidepressant.^b^				No
QI 32	Percent of care recipients who received an antianxiety and/or hypnotic medicine.^b^				No
QI 33	Percentage of care recipients who received chronic opioid medication.^b^				No
QI 34	Percentage of care recipients receiving opioid therapy who were screened for opioid‐related adverse effects.				No
QI 35	Percentage of care recipients experiencing a high sedative load (sedative load score ≥3).^b^				No
QI 36	Percentage of care recipients who received at least one potentially inappropriate medicine (PIM).^b^				No
QI 37	Percentage of care recipients receiving an anticholinergic medicine.^b^				No
QI 38	Percentage of care recipients that concurrently used three or more psychotropic medicines.^b^				Yes
QI 39	Number of medicine incidents reported per 1000 resident days.^b^				No
QI 40	Percentage of care recipients with an emergency department presentation or hospitalization for a medicines‐related event.^b^				No
QI 41	Percentage of care recipients with four or more regular medicine administration times.^b^				Yes
QI 42	Percentage of care recipients with a medicines self‐administration assessment.				No
QI 43	Percentage of care recipients that are appropriately assessed for risk of medicines‐related falls, within 14 days of LTCF admission.^a^				No
QI 44	Percentage of care recipients at risk of falls who take a medicine associated with an increased risk of falls.^b^				No
QI 45	Percentage of care recipients that are appropriately assessed for risk of medicines‐related impairment of cognitive and/or physical function.				No
QI 46	Percentage of care recipients with diabetes who are treated with glucose‐lowering medicines that have a documented hypoglycaemia management plan.				No
QI 47	Percentage of care recipients for whom a pharmacist has provided education to that person or their family member/proxy.				No
QI 48	Percentage of care recipients who were satisfied with the information provided about medicines.				No
QI 49	Percentage of care recipients who were involved as much as they wanted in decisions about medicines.				No
QI 50	Percentage of care staff receiving education about quality use of medicines.				No
QI 51	Percentage of care recipients for whom care staff were instructed by the pharmacist on how to monitor for the effectiveness and potential adverse effects of medicines.				No
QI 52	Percentage of care recipients at the end of life (or their family members/proxies) who receive information about medicines for managing end of life symptoms.				No
QI 53	Percentage of care recipients who were prescribed an opioid for pain during the last week of life.^a^				No
QI 54	Percentage of care recipients who were prescribed an anxiolytic/hypnotic medicine during the last week of life.^a^				No
QI 55	Percentage of care recipients who were prescribed a medicine for terminal respiratory secretions (death rattle) during the last week of life.^a^				No
QI 56	Percentage of care recipients who were prescribed an anti‐nausea drug during the last week of life.^a^				No
QI 57	Percentage of care recipients who were prescribed an anticipatory medicine during the last week of life.^c^				Yes
QI 58	Percentage of LTCFs that have anticipatory medicines available on imprest for care recipients during their last week of life.				Yes

Abbreviation: DI, disagreement index; LTCF, long‐term care facility; MAC, medication advisory committee; QI, quality indicator.

^a^ The wording of the indicator was adjusted from the first to the second round. Dark green indicates that a median score of ≥7 was reached with agreement (deviation index [DI] ≤ I), bright green indicated that a median score of ≥7 was reached with 70% of the panellists giving it a high rating (score of ≥7) but a DI > 1. Red indicated a median score <7 or disagreement. If agreement was defined solely by the DI criterion, QI 1 and QI 6 would not have reached agreement across all three categories.

^b^ QIs for which lower values are preferable, for the other indicators higher values indicate better quality.

c Anticipatory medicines are defined as ‘injectable or sublingual medications prescribed to a person with a life limiting illnesses. Availability of specific anticipatory medicines and other outlined medication classes in the QI list may vary in different countries and could be defined in accordance with local regulatory requirements and subsidized medicines programs.

**TABLE 5:** Quality indicators (QI) ratings received in the first and second Delphi round.

		Round 1							Round 2									
QI	Type	Importance		Feasibility		Amenability		Result	Importance			Feasibility			Amenability			Selected
		Mdn	DI	Mdn	DI	Mdn	DI		Mdn	DI	% ≥ 7^a^	Mdn	DI	% ≥ 7^a^	Mdn	DI	% ≥ 7^a^	
Domain—governance and leadership																		
QI 1	S	8	2.45	8	0.34	7	1.97	Adjust	8	0.26	88	8	2.53	100	7	0.56	83	Yes
QI 2	S	8	0.51	8	0.36	7	0.99	Agreement	—	—	72	—	—	84	—	—	67	Yes
QI 3	S	8	0.32	8	0.61	7	0.71	Agreement	—	—	88	—	—	72	—	—	67	Yes
QI 4	P	8	0.37	6	0.92	7	0.57	Agreement	—	—	88	—	—	48	—	—	75	No
QI 5	P	7	0.86	7	−0.52	7	0.57	Agreement	—	—	54	—	—	58	—	—	70	Yes
QI 6	P	—	—	—	—	—	—	Add QI	7	0.38	79	8	0.60	70	7	1.09	70	Yes
Domain—multidisciplinary clinical care and pharmacist services																		
Medication reviews and reconciliations																		
QI 7	P	9	0.40	7	0.79	8	2.40	Disagreement	9	0.18	92	7	0.61	71	8	0.22	96	Yes
QI 8	P	9	0.38	7	0.56	8	0.33	Agreement	—	—	87	—	—	70	—	—	87	Yes
QI 9	P	9	0.21	9	2.54	9	0.20	Adjust	9	0.16	100	9	0.36	83	9	0.16	100	Yes
QI 10	P	8	0.38	6	−0.27	7	1.47	Adjust	8	0.23	96	6	2.00	42	7	1.12	63	No
QI 11	P	7	2.19	6	1.65	7	0.91	Disagreement	8	0.68	92	7	1.09	54	7	0.40	79	No
QI 12	P	8	0.55	7	0.62	8	2.45	Adjust	8	0.40	83	7	0.40	83	8	0.47	88	Yes
QI 13	P	8	2.40	6	1.14	7	0.82	Disagreement	7	1.14	67	7	0.89	52	7	0.61	67	No
QI 14	P	7	0.87	6	1.10	7	0.87	Adjust	8	0.42	83	7	0.61	58	7	0.41	83	Yes
QI 15	P	7	2.28	5	2.44	6	0.88	Disagreement	7	0.82	71	5	1.25	25	6	0.86	42	No
QI 16	P	7	2.19	6	1.66	7	0.75	Disagreement	7	1.11	67	5	0.95	17	7	0.36	79	No
QI 17	O	7	0.86	6	1.49	5	0.82	Disagreement	7	0.51	71	6	1.22	30	5	1.05	21	No
QI 18	P	9	0.53	7	1.14	6	2.19	Disagreement	9	0.20	92	7	1.70	65	7	1.02	54	No
QI 19	P	—	—	—	—	—	—	Add QI	7	0.78	58	7	1.23	61	7	1.13	61	No
QI 20	P	—	—	—	—	—	—	Add QI	8	0.39	83	7	0.32	83	7	0.60	83	Yes
QI 21	P	—	—	—	—	—	—	Add QI	8	0.32	88	6	1.01	46	7	0.93	65	No
Collaboration between general practitioners and pharmacists, and other health care professionals																		
QI 22	P	5	1.35	4	0.75	5	0.82	Discard	—	—	—	—	—	—	—	—	—	No
QI 23	P	6	−0.55	5	1.18	6	0.88	Discard	—	—	—	—	—	—	—	—	—	No
Pharmacist services provided in the long‐term care facility																		
QI 24	P	7	2.28	6	2.22	5	1.52	Disagreement	6	0.95	50	6	1.23	46	5	0.93	21	No
QI 25	S	—	—	—	—	—	—	Add QI	7	0.90	58	8	0.62	83	7	0.84	61	Yes
Domain—medicines specific quality indicators																		
Antimicrobial use																		
QI 26	P	8	0.97	9	0.36	7	1.21	Disagreement	8	0.98	58	8	0.50	92	6	1.08	33	No
QI 27	P	6	0.83	6	1.19	5	1.37	Discard	—	—	—	—	—	—	—	—	—	No
QI 28	P	8	2.52	7	1.30	5	1.30	Disagreement	8	0.28	83	6	1.09	46	5	1.36	38	No
QI 29	P	8	0.63	6	0.95	5	1.00	Agreement	—	—	65	—	—	44	—	—	39	No
Vaccinations for care recipients																		
QI 30	P	9	2.57	8	0.37	8	0.61	Disagreement	9	0.23	96	8	0.35	88	8	0.45	79	Yes
Antidepressant use																		
QI 31	P	7	2.35	8	2.45	6	−0.48	Disagreement	7	1.15	54	8	0.40	83	5	1.79	25	No
Hypnotics/anxiolytic use																		
QI 32	P	9	0.34	8	0.61	7	0.97	Agreement	—	—	87	—	—	70	—	—	48	No
Opioid use																		
QI 33	P	9	0.36	8	0.63	6	0.00	Agreement	—	—	87	—	—	65	—	—	44	No
QI 34	P	8	0.79	5	−0.31	6	0.00	Agreement	—	—	65	—	—	22	—	—	35	No
Cumulative medication exposure																		
QI 35	P	8	0.60	7	2.19	5	1.87	Disagreement	8	0.40	83	7	1.92	58	5	1.05	29	No
QI 36	P	7	2.35	6	1.29	6	0.00	Disagreement	7	0.74	71	6	1.74	46	6	1.15	25	No
QI 37	P	7	0.76	6	−0.52	5	1.16	Disagreement	7	0.42	79	7	0.92	58	6	0.96	29	No
QI 38	P	8	0.30	7	2.35	6	0.91	Disagreement	8	0.30	96	7	0.50	75	7	0.70	54	Yes
Medication incidents and mediation‐related hospitalizations																		
QI 39	O	7	−0.91	7	−0.66	5	−0.45	Agreement	—	—	73	—	—	50	—	—	36	No
QI 40	O	8	0.38	6	1.85	6	0.00	Disagreement	8	0.35	88	6	1.58	44	6	1.20	33	No
Medication administration																		
QI 41	P	7	0.62	7	0.80	7	0.86	Agreement	—	—	59	—	—	64	—	—	59	Yes
QI 42	P	5	1.69	6	−0.31	5	−0.35	Discard	—	—	—	—	—	—	—	—	—	No
Risk assessment and prevention																		
QI 43	P	8	0.36	6	1.39	7	1.15	Adjust	8	0.32	92	6	1.91	46	7	1.07	67	No
QI 44	P	7	2.19	7	2.19	6	1.49	Disagreement	7	0.40	79	7	0.85	54	6	1.21	29	No
QI 45	P	8	0.39	5	1.21	7	0.00	Disagreement	8	0.35	88	5	1.36	46	5	0.91	46	No
QI 46	P	8	0.98	7	1.21	7	2.19	Disagreement	8	1.05	71	7	1.14	67	7	0.70	67	No
Domain—person‐centred care and communication																		
QI 47	P	7	1.29	6	1.24	7	0.85	Disagreement	7	1.00	71	6	1.12	25	7	0.85	63	No
QI 48	O	6	1.83	5	0.84	5	1.46	Discard	—	—	32	—	—	10	—	—	33	No
QI 49	P	7	0.78	5	0.99	6	1.86	Disagreement	7	0.44	79	5	1.09	17	6	1.03	21	No
Domain—staff education about quality use of medications																		
QI 50	P	8	0.93	7	2.19	7	1.15	Disagreement	8	2.46	79	7	1.32	58	7	0.72	71	No
QI 51	P	8	0.85	5	1.78	7	2.19	Disagreement	7	0.83	67	4	1.24	22	6	1.36	42	No
Domain—palliative and end of life medications																		
Communication with residents/families about medications for managing end‐of‐life symptoms																		
QI 52	P	8	0.62	5	1.74	6	1.25	Disagreement	8	0.55	88	6	1.36	33	6	1.07	50	No
Anticipatory medications																		
QI 53	P	7	2.28	7	1.20	5	−0.33	Adjust	7	1.68	58	7	0.40	79	5	1.27	25	No
QI 54	P	7	2.28	7	1.24	5	1.49	Adjust	7	1.88	54	7	0.54	75	5	1.16	21	No
QI 55	P	7	1.99	6	1.08	5	1.41	Adjust	6	1.45	50	6	0.89	46	5	1.31	33	No
QI 56	P	7	2.28	7	2.19	6	1.39	Disagreement	7	1.64	58	7	1.05	67	6	1.19	33	No
QI 57	P	—	—	—	—	—	—	Add QI	8	0.56	71	7	0.55	71	7	0.91	57	Yes
QI 58	S	—	—	—	—	—	—	Add QI	8	0.41	83	8	0.71	79	8	0.79	70	Yes

Abbreviations: DI, disagreement index; Mdn, median; O, outcome; P, process; QI, quality indicator; S, structure.

^a^ % > 7 refers to the percentage of panellists who gave a score of 7 or above. P, S, O, Mdn, and DI calculated as interpercentile range (difference between 30th and 70th percentile)/interpercentile range adjusted for symmetry) DI ≤ 1 indicates agreement.

### 3.3 Round 2

There were 42 QIs (process QI *n* = 36, structure QIs *n* = 3 and outcome QIs *n* = 3) presented in the second round (Table 5 and Figure 1). Of these, 12 QIs received high ratings across all three criteria with agreement and were selected (Table 4). Combined with the 5 QIs selected in Round 1, a total of 17 QIs (process *n* = 12, structure *n* = 5 and outcome *n* = 0), were rated high with agreement, meeting the selection criteria (outline in Table 2) for all three rating criteria. For the time frame of receiving at least one comprehensive medication review (QI 9), panel members had to select in the second round between 6 months (selected by 67%) and 12 months (selected by 33%). Among the six domains, most of the selected QIs (*n* = 7) fell within the multidisciplinary clinical care and pharmacist services domain, followed by governance and leadership domain (*n* = 5). Three QIs of the medicine‐specific domain and two QIs from the palliative and end‐of‐life medications domain were selected. None of the QIs from the person‐centred care domain met the selection criteria (Tables 4 and 5).

### 3.4 Final rating of QIs across rating criteria

Of the 58 QIs presented across the two Delphi rounds, none received a low rating (score of 1–3) on any of the three rating criteria. Overall, QIs received higher scores for importance than for feasibility or amenability to change. Based on the final ratings (using Round 2 scores for the QIs reassessed and Round 1 scores for those not re‐evaluated), 51 QIs (88%) received a high rating (score of 7–9) for importance, of which 45 QIs reached agreement. For feasibility, 34 QIs (59%) received a high score, of which agreement was reached for 27 QIs. Lastly, for amenability to change, 30 QIs (52%) received a high score, of which 25 reached agreements. Visual inspection of the median rating scores by panellists' group (Table S2) revealed similar rating patterns between medical practitioners, registered pharmacists and academics for importance and amenability to change. For feasibility, small differences emerged, with medical practitioners tending to assign lower scores for selected QIs, while academics tended to assign higher scores compared with pharmacists (see Table S2).

## 4 DISCUSSION

This two‐round modified Delphi study identified 17 QIs related to the safe and effective use of medications and pharmacist services as important, feasible to measure in LTCFs and amenable to change through pharmacists' clinical practice in LTCFs. The identified QIs target areas relating to comprehensive medication reviews and MACs and use of specific medications, such as anticipatory medications, and influenza vaccinations. Almost all QIs examined (*n* = 51 QIs, 88%) were deemed highly important by the experts, demonstrating the clinical relevance of the broader indicator set for Australian and international quality monitoring programmes in LTCFs beyond those ultimately selected. However, feasibility and amenability to change were often rated lower. This suggests that perceived or actual systemic barriers may hinder the implementation of relevant QIs for population‐level use in Australian LTCFs.

Six of the 17 selected QIs focused on medication reconciliation and/or review, highlighting their perceived value in the Australian context. Several countries have government‐funded programmes to support pharmacist‐led medication reviews in LTCFs, such as the Residential Medication Management Review (RMMR) service model in Australia and the MEdsCheck LTC programme in Canada^30^ to resolve medication‐related problems.^10^ Care transition points are recognized as high‐risk periods, often due to medication discrepancies, with medication‐related adverse events estimated to occur in around 20% of transitions between hospitals and LTCFs.^31^, ^32^ Medication reconciliation and review related QIs are already used in population‐level monitoring programmes internationally to assess whether reviews are conducted annually^33^, ^34^ and at key care transition points.^35^ Although existing QIs for assessing medication review frequency internationally do so annually, Delphi panellists recommended evaluating review frequency at least six‐monthly. However, the feasibility of more frequent medication reviews may be constrained by funding models.^36^ For instance, under Australia's RMMR model in LTCFs, reviews are funded following a GP referral at a minimum of biennially unless clinical circumstances have changed, whereas the ACOP model does not restrict medication review frequency. However, two thirds of our panellists recommended that reviews be monitored at least six monthly given that ACOPs work in LTCFs at least half a day per week. Accordingly, the proposed timeframes in these QIs primarily align more with clinical judgement and the ACOP model of care in Australian LTCFs. These timeframes may require adaptation when applied in other countries or funding contexts and will also be investigated during our planned implementation study. Additionally, assessing timely medication reconciliation at care transition points was suggested to assist in prioritizing medication reviews for residents. Situation and medication specific QIs that assess whether pharmacist‐led medication reviews were conducted in response to (i) a medication incident or (ii) regular psychotropic medication use were also rated highly across all three criteria, reflecting key areas of concern in LTCFs.

The governance and leadership domain included five QIs that met our selection criteria, focusing on medication governance committee activities, such as frequency, composition and review of education needs, that pharmacists can initiate in LTCFs. However, panellists emphasized the impact of funding of health professional's time on the amenability to change of governance QIs. In 2021, the Royal Commission into Aged Care Quality and Safety in Australia investigated systemic issues of public concern in LTCFs and highlighted the importance of an effective clinical governance system.^14^ Although organizational governance is covered by Australian LTCF quality standards, there is a noticeable gap in QIs that target clinical governance, organization culture and education needs related to medication use in LTCFs. Hence, further work is needed to support the establishment and monitoring of multidisciplinary clinical governance structures to support optimal medication practices.

A diverse range of QIs targeting individual or combination medication use were identified in our systematic review,^18^ but only five medication‐specific QIs met all our selection criteria. Two of the five selected QIs focus on the use and/or availability of anticipatory medications. The administration of anticipatory medications can reduce emergency hospital transfers at end of life and alleviate pain and distress.^37^ The QI ratings and qualitative feedback from the Delphi panel suggest that pharmacists can support effective use of anticipatory medications at end of life, not only through stock management but also via education.^38^ Other selected QIs included cumulative exposure to three or more psychotropic medications, resident influenza vaccination rates and having four or more medication administration times per day. Minimizing the number of medication administration times, to reduce burden for residents and enable staff to shift time to other care activities in the LTCFs, can be effectively reduced by pharmacists and medication practitioners using standardized simplification tools.^39^, ^40^ Psychotropic medication use is often linked with significant adverse effects and remains a key area of concern in LTCFs.^41^ In Australia, the National Aged Care Mandatory QI programme currently monitors polypharmacy and antipsychotics use, a specific class of psychotropic medications. Since the implementation of the national programme in mid‐2021, antipsychotic use decreased from 21.6% to 17.3% in 2025.^42^ However, the use of other psychotropic medications, such as antidepressant use,^43^ has been increasing, raising concerns about chemical restraint, underscoring the potential importance of monitoring psychotropic medication use within LTCFs.^44^, ^45^

Despite receiving high ratings for importance and feasibility, medication‐specific QIs related to chronic opioid use, anxiolytics and hypnotic use were perceived as less amenable to change through pharmacist interventions, highlighting the multifaceted challenges in altering prescribing behaviours. Deprescribing is often a complex and prolonged process, which is linked with barriers such as patient understanding, the management of withdrawal symptoms as well as unintended harms.^46^ While a multidisciplinary approach and patient and prescriber education, in which pharmacists play a key role, can support appropriate prescribing and deprescribing efforts,^46^, ^47^ multiple factors must be considered when monitoring high‐risk medication use such as opioids and sedatives. This includes the risk of unintended consequences such as pain not being assessed or analgesics not being charted when pain is present. Integrated models, such as stewardship programmes, that involve interprofessional collaboration coupled with supportive regulatory frameworks, are important in shifting prescribing patterns and enhancing amenability to change.^47^ Similarly, although outcome indicators related to medication‐related hospitalizations and incidents were rated as important, they were perceived as less feasible to collect in the LTCF and/or amenable to change. Unlike process or structure indicators, outcome measures are often influenced by multiple factors beyond direct care delivery, meaning that changes in clinical pharmacist practices may not directly translate into immediate improvements, particularly for relatively infrequent events.^18^, ^48^ There are also challenges with collecting data manually from LTCFs relating to hospitalization outcomes, and these indicators may be suitable to monitor using integrated health and aged care data platforms.

Medication management in LTCFS requires a strong focus on person‐centred care, ensuring that treatment aligns with the resident's values, needs and preferences. Although medication safety and effectiveness QIs should contribute to a better care experience, there is a growing recognition of the need for QIs that explicitly assess the care recipient's perspective. In this Delphi study, person‐centred QIs were perceived as important, but were ultimately discarded due to feasibility concerns. This likely stems from the perceived reporting burden of incorporating additional measurement processes into LTCFs, as well as challenges that arise with collecting data from residents with cognitive impairment, as noted in qualitative comments from multiple Delphi participants. The adoption of electronic medication management systems can ease documentation, streamline interprofessional communication and support medication reconciliation, increasing the feasibility of QIs related to medication use and documentation. In this context, the Australian Government is currently supporting the rollout of an electronic national residential medication chart (eNRMC). Notably, although the use of eNRMC is not yet mandatory, Australian LTCFs that adopt the new on‐site pharmacist model are required to transition to an eNRMC if one is not already in place. A recent survey of South Australian LCTFs found that 81% of the assessed facilities employed an eNRMC and 74% of the LTCFs used an electronic clinical documentation system.^49^ However, these systems are not always interoperable and implementation of person‐centred QIs requires extra effort beyond implementing an eNRMC and/or clinical care system. Australia has recently implemented two QIs to assess consumer experience and quality of life that are directly collected from care recipients (or proxies if the recipient is unable to complete the survey). This demonstrates that person‐centred QIs can be measured on a population level, and it may be feasible to add additional person‐centred QIs, although this should be balanced with our Delphi participant's concerns relating to data collection burden for LTCFs.

It is important that identified QIs, regardless of whether they were selected based on rating thresholds, are mapped onto a comprehensive quality framework to ensure that the full spectrum of medication management, including person‐centred care, is captured in future evaluation efforts. The presented QIs serve as a valuable starting point to assess variation in practices that may influence the quality of care people receive. However, further work is needed to evaluate the viability and effectiveness of the QIs. Key aspects, such as appropriate reporting frequency, the application of risk adjustment and the overall suitability of the indicators, for benchmarking require further refinement so the QIs can be evaluated on the ground in LTCFs and using real‐world datasets to further investigate feasibility.

### 4.1 Strengths and limitations

Strengths include access to the expertise of a large, multidisciplinary expert panel that evaluated the QIs using three selection criteria. This approach exceeds the norms of previous Delphi studies investigating health care QIs, which typically include smaller panels (median of 17) and few selection criteria (<10% apply three selection criteria).^50^ The list of QIs presented to the panel was informed by a timely and comprehensive systematic review, encompassing a broad range of medication management QIs.^18^ Although the Delphi panel did not directly include consumers, due to the technical nature of many QIs, which often require pharmacological expertise, a consumer advisory group was regularly consulted (quarterly) throughout the project. Consumers were also involved in a pre‐Delphi consumer workshop that informed the selection of QIs for the Delphi and provided feedback in the health professional workshop. Following discussion with our consumer advisory group, the group advised not to include consumers in the Delphi rounds, but they continue to be consulted in the next project stages in which suitable QIs have been mapped onto a comprehensive, person‐centred quality framework.^51^

Study limitations include that the Delphi was conducted online, limiting the interaction and discussions among experts. The QIs were not presented in a counterbalanced order, meaning that the sequence presented may have influenced the experts' ratings. Additionally, detailed QI measurement specifications or risk adjustments were not included. Experts in Australia rated the QIs within the context of Australian LTCFs, which is a strength for the evaluation of the new Australian LTCF ACOP programme but needs to be taken into consideration when considering QI implementation in other countries. This is particularly relevant when interpreting the feasibility of QIs, which are dependent on local healthcare infrastructure, clinical guidelines and system‐level factors that may vary internationally. Furthermore, perceived feasibility may differ from actual feasibility when QIs are implemented in practice. Moreover, the assessment of amenability focused on pharmacists' scope of practice in Australia, and the role of the pharmacist and other healthcare professionals in Australia may differ from other countries. Nevertheless, the list of rated QIs remains internationally relevant, especially in terms of their importance rating. Given that Australia is among the first countries to embed pharmacists on‐site in LTCFs, in countries that adopt similar models, the amenability of these indicators to pharmacist‐led interventions may be more closely aligned with our findings. The highly rated QIs identified in this Delphi have a direct path to implementation and have been mapped onto a quality framework,^51^ in preparation for further refinement and testing using real‐world LTCF data as part of the PHARMA‐Care programme.

## 5 CONCLUSION

This Delphi study identified 17 QIs that are considered important, feasible to measure and amenable to change through clinical practice, to assess and monitor safe and effective medication use in LTCFs. However, to capture the complexity of medication management in LTCFs and to enhance person‐centred care, multiple factors and domains need to be considered. Mapping the identified QIs onto a comprehensive quality framework is an important next step to evaluate medication safety and effectiveness more broadly, to ultimately enhance the quality of care and outcomes for individuals accessing LTCFs.

## AUTHOR CONTRIBUTIONS

Janet K. Sluggett conceptualized the study. All authors contributed to the study design. Daria S. Gutteridge, Sara Javanparast, Janet K. Sluggett and Annabel H. Calder developed the e‐Delphi. Janet K. Sluggett and Sara Javanparast recruited the panel members. Daria S. Gutteridge analysed the results, and all authors discussed the results. Daria S. Gutteridge drafted the manuscript, and all authors critically revised the initial manuscript draft for important intellectual content. All authors read and approved the final manuscript.

The authors confirm that the principal investigator for this paper is Janet K. Sluggett and that this study did not involve direct clinical care.

## CONFLICT OF INTEREST STATEMENT

J. K. S. is a non‐executive director of Southern Cross Care SA, NT, Victoria (aged care provider organization). The other authors declare no perceived or actual conflicts of interest.

## Supporting information


**Table S1.** Definition of quality indicators (QI) including the numerator and denominator.
**Table S2.** Quality indicator (QI) ratings my panellist's profession (*N* > 5), note some panellists counted towards multiple professions.

## Data Availability

The dataset generated during the current study is not publicly available to main the anonymity of the panel members. However, summary data may be requested from the corresponding author.
